# Systemic immune-inflammation index (SII) and the risk of all-cause, cardiovascular, and cardio-cerebrovascular mortality in the general population

**DOI:** 10.1186/s40001-023-01529-1

**Published:** 2023-12-09

**Authors:** Huan Wang, Huiyong Nie, Gang Bu, Xiaoning Tong, Xiaofang Bai

**Affiliations:** 1 Department of Pain Medicine, The First Affiliated Hospital of Xi’an Jiaotong University, Xi’an , 710061 China; 2 Department of Clinical Laboratory, The First Affiliated Hospital of Xi’an Jiaotong University, Xi’an, 710061 China; 3 The Department of Ultrasound Medicine, The First Affiliated Hospital of Xi’an Jiaotong University, No. 277, Yanta West Road, Xi’an, 710061 Shaanxi Province China

**Keywords:** NHANES, Systemic immune-inflammation index SII, All-cause mortality, Cardiovascular mortality, Cardio-cerebrovascular mortality

## Abstract

**Background:**

An elevated systemic immune-inflammation index (SII) is associated with higher mortality in patients with coronary artery disease and other diseases. However, the potential of SII for predicting mortality in the general population has been underexplored. Therefore, this study aimed to analyze the relationship between the SII and all-cause, cardiovascular disease, and cardiocerebrovascular disease mortality in the general population.

**Methods:**

This study involved 26,855 participants (≥ 18 years) from the National Health and Nutrition Examination Survey 1999–2014 who were grouped according to the SII tertiles. Survival differences between the groups were analyzed using log-rank tests and Kaplan–Meier plots. Furthermore, multivariate Cox regression and restricted cubic spline analyses were used to examine the relationship between the SII and all-cause, cardiovascular, and cardio-cerebrovascular mortality.

**Results:**

Overall, 1947 (7.425%) participants died following an average follow-up of 87.99 ± 54.04 months. Among these, 325 (1.210%) deaths were related to cardiovascular diseases and 392 (1.459%) to cardio-cerebrovascular mortality. Kaplan–Meier analysis revealed statistically significant differences in all-cause, cardiovascular, and cerebrovascular mortality between the SII tertiles (log-rank test: all *P* < 0.001). Multi-adjusted models showed that participants in the highest tertile of SII had a higher risk of death from all-cause (hazard ratio [HR] = 1.48, 95% confidence interval [CI] 1.48–1.48) and cardiovascular mortality (HR = 1.60, 95% CI 1.60–1.61) compared with those in the lowest tertile. In addition, the restricted cubic spline curve indicated a nonlinear association between SII and all-cause mortality (P < 0.001), with threshold value of SII at 18.284. There was a 15% decrease in the risk of all-cause mortality for each twofold change in SII on the left flank (HR = 0.85, 95% CI 0.69–1.05) and a 42% increase (HR = 1.42, 95% CI 1.23–1.64) on the right flank of the inflection point. In addition, the risk of cardiovascular mortality increased nonlinearly by 39% per twofold change in SII (HR = 1.39, 95% CI 1.07–1.81). There was also a nonlinear increase in the risk of cardio-cerebrovascular mortality per twofold change in SII (HR = 1.29, 95% CI 1.00–1.66).

**Conclusions:**

In the general population, the SII was significantly associated with all-cause, cardiovascular, and cardio-cerebrovascular mortality, regardless of the established risk factors.

**Supplementary Information:**

The online version contains supplementary material available at 10.1186/s40001-023-01529-1.

## Introduction

Nearly one-third of all deaths worldwide are caused by cardiovascular disease (CVD) [1, 2]. In the United States (US), this accounts for 17% of all health expenditures [2, 3]. Therefore, effective mortality prediction tools are essential for preventing and treating CVD as soon as possible [4]. The burden of CVD and disability-adjusted life years is largely attributed to stroke and ischemic heart disease [5], and atherosclerosis is the major cause of these diseases [6]. Atherosclerosis is associated with thrombosis, oxidative stress, and endothelial damage. [7, 8]. Recent research has shown that the immune system has a profound relationship with inflammation during the development of atherosclerosis [8, 9]. The cells of the immune system comprise lymphocytes, neutrophils, monocytes, and macrophages, which play different roles in atherosclerosis development. For example, neutrophils activate macrophages, promote monocyte recruitment, and initiate cytotoxicity at various stages of atherosclerosis. In contrast, lymphocytes regulate inflammation and reduce atherosclerosis [10–12]. In addition, platelets adhere to vessel walls, aggregate leukocytes, and initiate atherosclerosis before invading plaques [13–16].

Recently, various cost-effective and verified inflammatory and immune indicators have been examined. The neutrophil–lymphocyte ratio (NLR) and platelet-lymphocyte ratio (PLR) offer better predictions of CVD outcomes than single cells [16—18]. A new systemic immune-inflammation index (SII), calculated as (neutrophil × platelet)/lymphocyte, has been introduced for CVD, which provides a more comprehensive reflection of immunity and inflammation [19, 20]. SII can be easily obtained by routine whole blood count test (the most commonly used clinical test). This marker was proposed by Hu et al. [21]. In the field of cancer, SII has a better predictive value for prognosis compared to other inflammatory factors [22, 23]. Studies have also indicated that SII may be crucial in CVD prognosis and mortality [24].

In some studies, the SII has been associated with CVD risk. However, inconsistent results have been obtained, highlighting the importance of early detection and intervention for CVD. Therefore, we aimed to investigate the clinical significance of the SII in predicting all-cause, cardiovascular, and cardio-cerebrovascular mortality in the adult population. Further, we aimed to assess the effectiveness of the SII as an affordable and accessible CVD risk indicator.

## Methods

### Study population

**Fig. 1:**
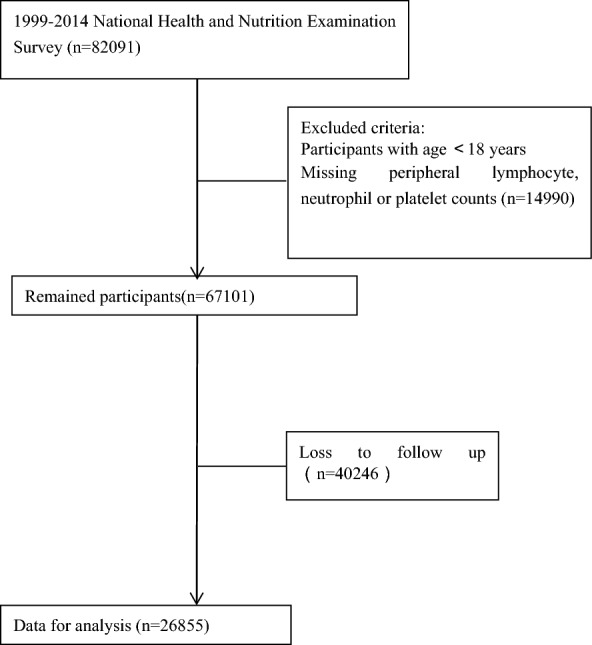
Flow chart of the participants

The population for this study was from the National Health and Nutrition Survey (NHANES) survey. Informed consent was obtained from all participants, and the NCHS Ethics Review Board approved the study protocol. Additional file 1 (Table S1) that shows the baseline characteristics of the included population and the follow-up missing population.  Overall, 82,091 participants from the surveys conducted between 1999 and 2014 were selected for this study. Exclusion criteria were as follows: (a) individuals aged < 18 years (n = 0); (b) individuals whose peripheral lymphocyte, neutrophil, and platelet counts were not available (n = 14,990); and c) individuals who were lost to follow-up (n = 40,246). Consequently, the final analysis was conducted on 26,855 participants. Figure 1 shows a flowchart of the selection of the study population.

### Exposure

The NHANES Laboratory/Medical Technologists Procedures Manual (LPM) describes the procedures for collecting and processing blood specimens. The complete blood count (CBC) parameters were determined using the Coulter^®^ method for counting and sizing, as well as an automatic mixing and dilution device for sample processing. Hemoglobinometry was performed using a single-beam photometer, while VCS technology was used for differential analysis of WBCs. The three simultaneous measurements conducted included individual cell volume (V), high-frequency conductivity (C), and laser light scattering (S). The Scattergrams plot the cells based on these three parameters.

Under established procedures, all blood specimens were withdrawn in the morning following a 9-h fast. CBC for all blood samples was obtained using Beckman Coulter MAXM. Three simultaneous measurements, comprising V, C, and S were used to sort the neutrophils and lymphocytes from the white blood cells. The number of thrombocytes was determined by multiplying the Plt histogram by the calibration constant, and expressing it as n × 10^3^ cells/µL. The SII was calculated as (neutrophils × platelets)/lymphocyte [21].

### Outcomes

The NCHS data linkage website provides detailed information on mortality status derived from the NHANES-linked National Death Index records [25]. Study outcomes, including all-cause, cardiovascular, and cardio-cerebrovascular mortality, were defined according to the 10th Revision of the International Classification of Diseases (ICD-10) [26]. All -cause death is the primary observation outcome event, while cardiovascular death and cardio-cerebrovascular death are secondary observation outcome events. The follow-up period was recorded from December 31, 2015, until the date of participation.

### Covariates

Demographic information, such as age, sex, and ethnicity, was collected through interview questionnaires. Participants who consumed 12 or more drinks in the last 12 months were classified as drinkers, while those who had smoked at least 100 cigarettes in their lifetime were classified as smokers [27]. The questionnaires also asked participants to self-report medical comorbidities such as hypertension, heart failure, diabetes mellitus, coronary heart disease, cancer, and stroke. Other covariates included hemoglobin, platelets, low-density lipoprotein cholesterol (LDL-C), high-density lipoprotein cholesterol (HDL-C), triglycerides, albumin, glycohemoglobin, blood urea nitrogen, creatinine, uric acid, cholesterol, and glucose levels.

### Statistical analysis

The study participants were classified into three groups based on their SII tertile: low (< 391,809.52), medium (391,809.52–611,840), and high (> 611,840) SII groups. Continuous variables were expressed as means ± standard deviations, while categorical variables were expressed as frequencies with percentages. One-way ANOVA was used to analyze the differences in categorical or continuous variables among the SII tertiles. Kaplan–Meier plots and log-rank tests were used to determine the differential survival rates of all-cause, cardiovascular, and cardio-cerebrovascular mortality according to SII tertiles.

Multivariate Cox regression models were used to examine the relationship between the SII and all-cause, cardiovascular, and cardio-cerebrovascular mortality with hazard ratios (HRs) and 95% confidence intervals (CIs). Model 1 was unadjusted, and model 2 was adjusted for age, sex, ethnicity, educational level, marital status, smoking status, and drinking status. Model 3 was further adjusted for diabetes mellitus (DM), hypertension, heart failure (HF), coronary heart disease (CHD), stroke, cancer, body mass index (BMI), and levels of hemoglobin, glycohemoglobin, platelets, blood urea nitrogen, creatinine, uric acid, cholesterol, glucose, LDL-C, HDL-C, triglycerides, and albumin.

A restricted cubic spline (RCS) regression model was used to investigate the nonlinear relationship between the SII (per twofold change) and all-cause, cardiovascular, and cardiocerebrovascular mortality. The threshold point was determined for nonlinear relationships using a two-piece Cox regression model. Further investigation of the effect of the SII on death risk among men and women was conducted using sex-specific models. Empower(R) (X&Y Solutions, Inc., MA, USA) and Stata (version 14.0) were used for statistical analysis. Statistical significance was set at *P*-value < 0.05.

## Results

### Characteristics of participants at baseline

**Fig. 2:**
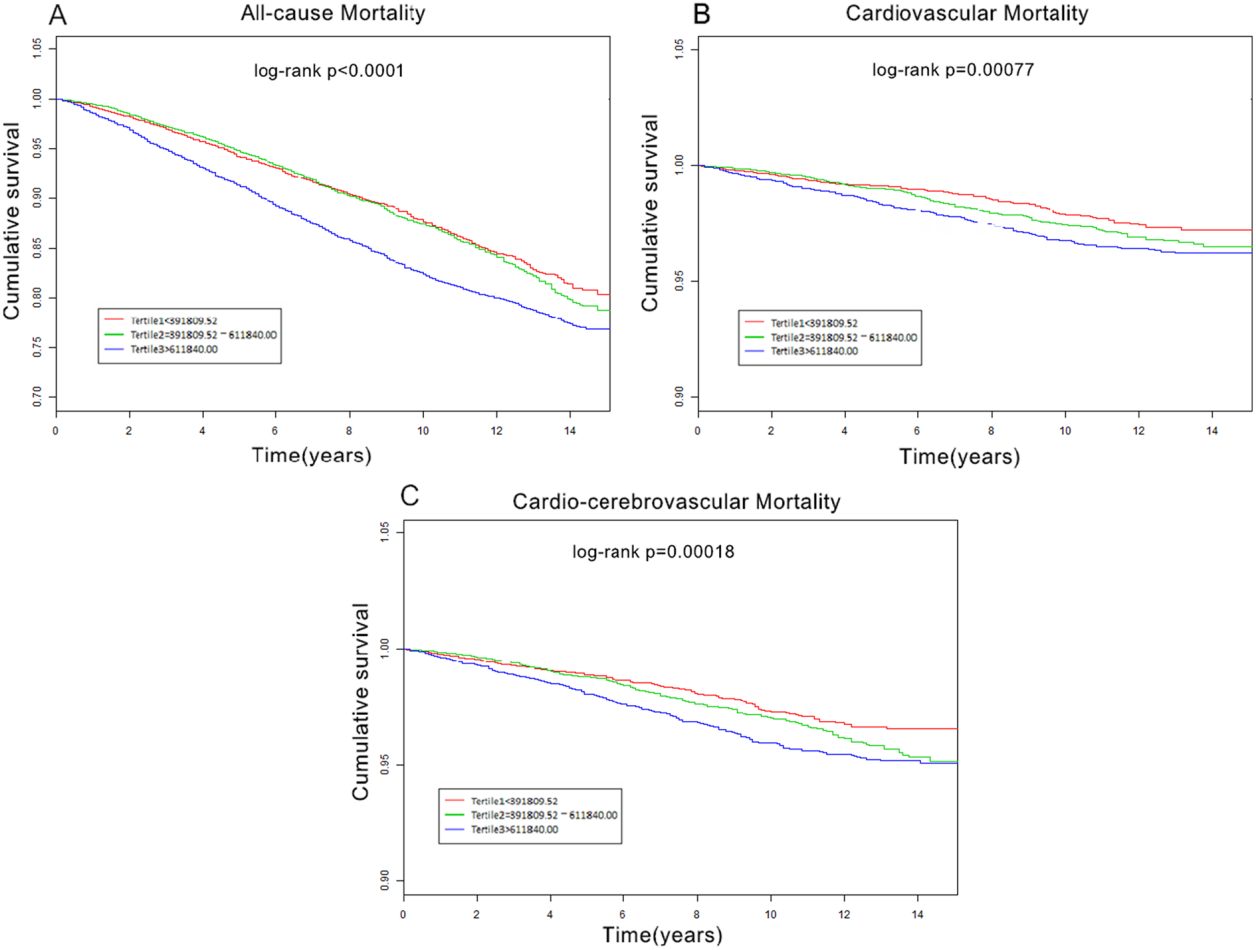
Kaplan–Meier curves for all-cause (**A**), cardiovascular mortality (**B**) and cardio-cerebrovascular mortality (**C**) according to SII tertiles. SII: systemic immune-inflammation index; (Platelet count × Neutrophils)/lymphocytes

**Fig. 3:**
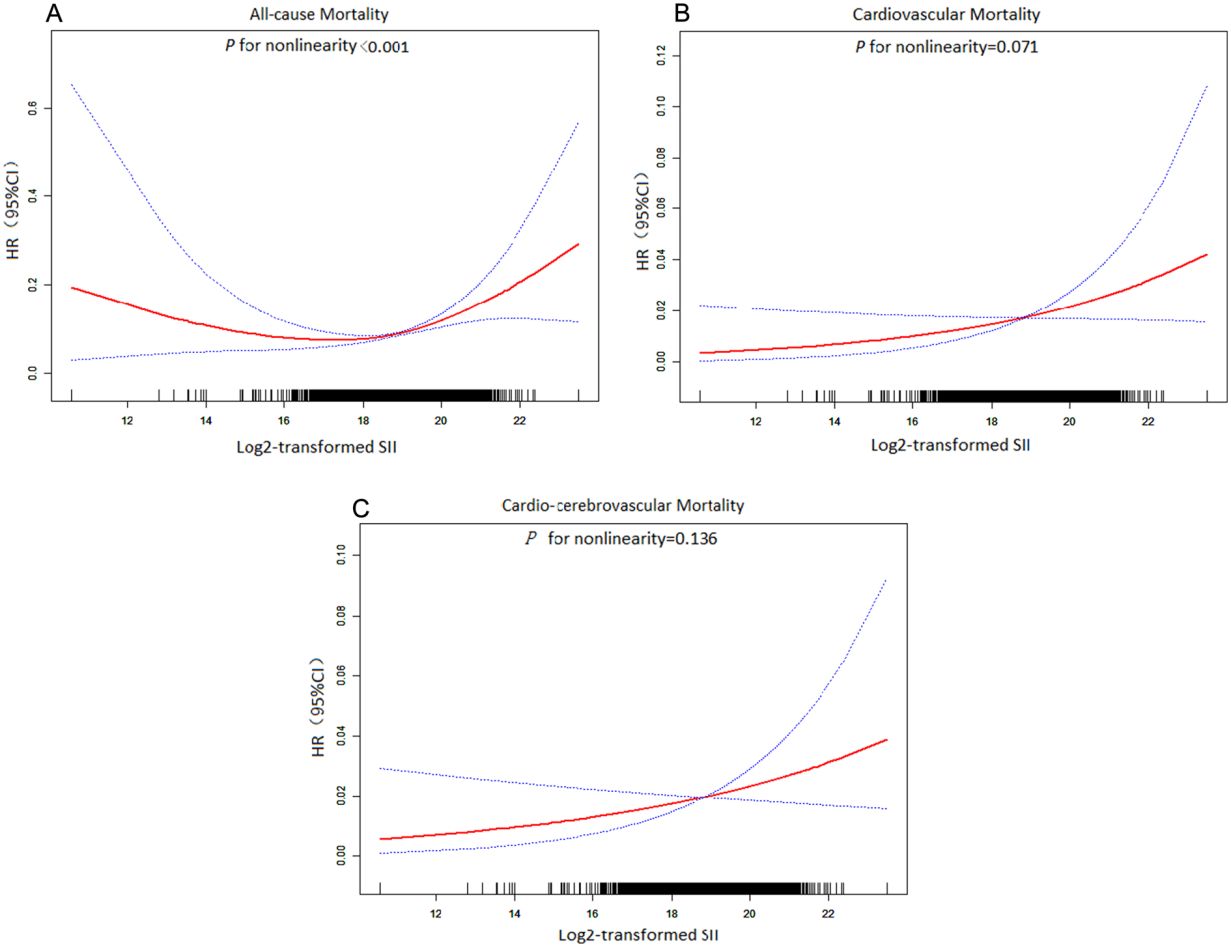
Restricted cubic spline curves of relations between SII with all-cause (**A**), cardiovascular mortality (**B**) and cardio-cerebrovascular mortality (**C**). Analysis was adjusted for age, gender, ethnicity, education, marital status, smoker, drinker, DM, hypertension, HF, CHD, stroke, cancer, BMI, hemoglobin, glycohemoglobin, platelets, blood urea nitrogen,uric acid, creatinine, cholesterol glucose, LDL-C, HDL-C, triglyceride, albumin. The solid and dashed lines symbolize the hazard ratios and corresponding 95% confidence intervals, respectively

Table 1 compares the baseline characteristics of the 26,855 participants included in this study. The participants had an average age of 45.674 ± 17.238 years, and included 48.268% male participants. The average follow-up period was 7.33 ± 4.50 years, during which 1995 (7.43%) deaths occurred, including 325 (1.210%) and 392 (1.459%) caused by cardiovascular and cardio-cerebrovascular mortality, respectively. Most baseline covariates were statistically significant, except for uric acid, blood urea nitrogen, and HDL-C levels (*P* < 0.05). Kaplan–Meier curves for all-cause mortality showed worse outcomes with increase in the SII (log-rank *P* < 0.001; Fig. 2A). The fully adjusted Cox regression model (Table 2) showed HRs (95% CI) of 1.018 and 1.479 for participants in the medium and highest tertiles, respectively, compared with those in the lowest tertile. Based on the RCS model (Fig. 3A), the association between the SII and all-cause mortality was nonlinear and U-shaped (*P* < 0.001).

**Table 1:** Baseline characteristics of the study population stratified by tertiles of SII value

Variable	Total (n = 26,855)	SII			*P* value
		< 391,809.52 n = 8951	391,809.52–611,840 n = 8951	> 611,840 n = 8953	
SII	571,361.23 ± 348,108.33	292,056.57 ± 71,128.54	493,189.16 ± 62,910.48	912,502.63 ± 394,147.47	< 0.001
Age, years	45.674 ± 17.238	45.059 ± 17.303	45.604 ± 16.704	46.319 ± 17.694	< 0.001
Male (%)	48.268	53.637	48.776	42.741	< 0.001
Ethnicity (%)					< 0.001
Non-Hispanic white	69.179	63.070	71.043	72.940	
Non-Hispanic black	10.879	16.362	9.121	7.590	
Mexican American	8.246	8.289	8.131	8.323	
Other race	11.697	12.279	11.704	11.147	
Education (%)					< 0.001
< High school	16.964	18.083	15.715	17.215	
High school	22.828	20.964	23.272	24.105	
> High school	56.628	56.871	57.728	55.263	
Other	3.580	4.083	3.285	3.417	
Body mass index, kg/m^2^	28.471 ± 6.599	27.701 ± 5.976	28.521 ± 6.401	29.137 ± 7.246	< 0.001
Marital status (%)					< 0.001
Never married	20.712	20.337	20.266	21.524	
Married	61.746	63.095	63.071	59.118	
Widowed	15.329	13.612	14.800	17.476	
Other	2.212	2.956	1.863	1.882	
Drinker (%)	11.339	11.076	10.325	12.633	< 0.001
Smoker (%)	45.260	42.896	44.575	48.172	< 0.001
Diabetes mellitus (%)	8.190	7.690	7.540	9.329	< 0.001
Hypertension (%)	29.719	27.280	29.620	32.093	< 0.001
Heart failure (%)	2.323	2.124	1.819	3.029	< 0.001
Coronary heart disease (%)	3.255	3.378	2.807	3.604	< 0.001
Stroke (%)	2.637	2.311	2.526	3.055	< 0.001
Cancer (%)	8.905	7.756	8.727	10.159	< 0.001
Hemoglobin, g/dL	14.320 ± 1.500	14.340 ± 1.428	14.410 ± 1.470	14.208 ± 1.587	< 0.001
Platelets, 10^3^/μL	254.704 ± 66.015	217.850 ± 50.711	252.425 ± 53.501	291.398 ± 70.382	< 0.001
Lymphocyte, 10^3^/μL	2.118 ± 1.018	2.382 ± 1.514	2.118 ± 0.644	1.872 ± 0.619	< 0.001
Neutrophil, 10^3^/μL	4.338 ± 1.719	3.121 ± 0.975	4.155 ± 1.106	5.661 ± 1.857	< 0.001
Triglyceride, mmol/L	1.679 ± 1.397	1.612 ± 1.323	1.711 ± 1.486	1.708 ± 1.366	< 0.001
Blood urea nitrogen, mmol/L	4.641 ± 1.901	4.640 ± 1.739	4.653 ± 1.797	4.631 ± 2.136	0.728
Cholesterol, mmol/L	5.063 ± 1.063	4.975 ± 1.065	5.109 ± 1.046	5.095 ± 1.073	< 0.001
Uric acid, μ mol/L	321.411 ± 83.162	321.776 ± 81.840	321.234 ± 81.974	321.255 ± 85.568	0.888
Creatinine, μ mol/L	78.541 ± 31.553	79.495 ± 31.037	78.001 ± 25.546	78.213 ± 37.142	0.003
LDL cholesterol, mmol/L	2.972 ± 0.905	2.904 ± 0.885	3.035 ± 0.906	2.979 ± 0.919	< 0.001
HDL cholesterol, mmol/L	1.369 ± 0.407	1.371 ± 0.409	1.362 ± 0.400	1.375 ± 0.410	0.148
Albumin, g/L	42.789 ± 3.354	43.119 ± 3.118	43.013 ± 3.135	42.250 ± 3.700	< 0.001
Glycohemoglobin	5.554 ± 0.891	5.544 ± 0.876	5.544 ± 0.883	5.573 ± 0.911	0.04268
All-cause mortality (%)	7.425	5.897	6.460	9.847	< 0.001
Cardiovascular mortality (%)	1.210	0.908	1.136	1.568	< 0.001
Cardio-cerebrovascular mortality (%)	1.459	1.100	1.371	1.885	< 0.001

Mean ± SD for: SII, age, body mass index, hemoglobin, albumin, cholesterol, triglycerides,uric acid, Blood urea nitrogen, creatinine, glycohemoglobin, HDL cholesterol, LDL cholesterol, Platelets, Lymphocyte, Neutrophil. *P* value was calculated by weighted linear regression model

% for: gender, race, educational level, marital status, smokers, drinkers, hypertention,diabetes mellitus,coronary heart disease,stroke,cancer,congestive heart failure. *P* value was calculated by weighted chi-square test

HDL-C high-density lipoprotein cholesterol, LDL-C low-density lipoprotein cholesterol, SII: (Platelet count × Neutrophils)/lymphocytes

Values are mean ± standardized deviation or number (%)

**Table 2:** Multivariate Cox regression models between SII with all-cause, cardiovascular and cardio-cerebrovascular mortality

	Tertile 1 HR	Tertile 2 HR (95%CI) *P*-value	Tertile 3 HR (95%CI) *P*-value
All-cause mortality			
Model 1	1.00	0.985 (0.984, 0.985)^*^	1.423 (1.422, 1.424)^*^
Model 2	1.00	1.037 (1.037, 1.038)^*^	1.422 (1.421, 1.423)^*^
Model 3	1.00	1.108 (1.107, 1.109)^*^	1.479 (1.477, 1.480)^*^
Cardiovascular mortality			
Model 1	1.00	1.132 (1.130, 1.134)^*^	1.489 (1.487, 1.491)^*^
Model 2	1.00	1.202 (1.201, 1.204)^*^	1.456 (1.454, 1.458)^*^
Model 3	1.00	1.328 (1.325, 1.332)^*^	1.602 (1.597, 1.606)^*^
Cardio-cerebrovascular mortality			
Model 1	1.00	1.124 (1.122, 1.125)^*^	1.471 (1.469, 1.472)^*^
Model 2	1.00	1.192 (1.190, 1.193)^*^	1.448 (1.446, 1.450)^*^
Model 3	1.00	1.212 (1.209, 1.215)^*^	1.396 (1.393, 1.400)^*^

CI Confidence interval, HR Hazard ratio, SII (Platelet count × Neutrophils)/lymphocytes

Model 1was adjusted for none. Model 2 was adjusted for age, gender, ethnicity, education, marital status, smoker, and drinker. Model 3 was further adjusted for DM, hypertension, HF, CHD, stroke, cancer, BMI, hemoglobin, glycohemoglobin, platelets, blood urea nitrogen, creatinine, cholesterol, glucose, LDL-C, HDL-C, triglyceride, albumin

^*^P < 0.00001

The results of the two-piecewise Cox regression analysis are shown in Table 3. To fit the models, the SII was log^2^-transformed because of its skewed distribution. The *P*-values for the logarithmic likelihood ratio test were < 0.001 for the one-line Cox regression model and the two-piecewise regression model. A threshold value of 18.284 was determined for the SII. On the left and right flanks of the inflection point, a twofold change in SII resulted in a 15% decrease (HR = 0.85, 95% CI 0.69–1.05) and a 42% increase (HR = 1.42, 95% CI 1.23–1.64), respectively (see Additional file 1).

**Table 3:** Threshold effect analysis of SII on mortality using the two-piecewise regression model

	All-cause mortality HR (95%CI) *P*-value	Cardiovascular mortality HR (95%CI) *P*-value	Cardio-cerebrovascular mortality HR (95%CI) *P*-value
Model I			
One line	1.204 (1.077,1.345)0.0011	1.218 (0.987,1.503) 0.0657	1.163 (0.955, 1.417) 0.1340
Model II			
Inflection value	18.284	18.297	18.308
< threshold value	0.851 (0.691, 1.047) 0.1274	0.926 (0.640,1.340) 0.6846	0.942 (0.657, 1.349) 0.7426
≥ threshold value	1.418 (1.229,1.635) < 0.0001	1.389 (1.066,1.810) 0.0150	1.288 (1.001, 1.658) 0.0494
P for log-likelihood ratio test	< 0.001	0.132	0.223

CI Confidence interval, HR Hazard ratio, SII (Platelet count × Neutrophils)/lymphocytes

SII was log2-transformed to fit the Cox regression model

Analysis was adjusted for age, gender, ethnicity, education, marital status, smoker, drinker, DM, hypertension, HF, CHD, stroke, cancer, BMI, hemoglobin, glycohemoglobin, platelets, uric acid, blood urea nitrogen, creatinine, cholesterol, glucose, LDL-C, HDL-C, triglyceride, albumin

### The association between SII and cardiovascular mortality

The Kaplan–Meier plot (Fig. 2B) showed that increased SII values were associated with reduced survival in CVD (log-rank *P* = 0.00077). When adjusted for all covariates (Table 2), the HR and CIs of cardiovascular mortality for those in the medium and highest tertiles were 1.33 (1.32–1.33) and 1.60 (1.60–1.61), respectively.

The RCS curve indicated a nonlinear association between SII and cardiovascular mortality (*P* for non-linearity = 0.071; Fig. 3B).

### The association between SII and cardio-cerebrovascular mortality

In the Kaplan–Meier plot for cardiocerebrovascular disease (Fig. 2C), the higher SII values were associated with reduced survival (log-rank *P* = 0.00018). After adjusting for all covariates (Table 2), the HR and 95% CIs of cardio-cerebrovascular mortality were 1.21 (1.21–1.22) and 1.40 (1.39–1.40) for individuals in the medium and highest tertiles, respectively.

Figure 3C shows that the SII was nonlinearly associated with cardio-cerebrovascular mortality (*P* for non-linearity = 0.136).

### Subgroup analysis

Table 4 shows that the HR and 95% CIs of cardiovascular mortality for women in the medium and highest tertiles were 3.906 (3.89–3.93) and 3.575 (3.56–3.595), respectively. In the same tertiles, the cardiovascular mortality HR and 95% CIs were 0.688 (0.68–0.69) and 1.24 (1.24–1.25), respectively.

**Table 4:** Stratified association between SII with all-cause, cardiovascular and cardio-cerebrovascular mortality by sex

	Tertile1 HR	Tertile2 HR (95% CI)	Tertile3 HR (95% CI)	*P*-value
				Trend
All-cause mortality				
Female	1.00	1.108 (1.106, 1.110)^*^	1.553 (1.550, 1.555)^*^	< 0.001
Male	1.00	1.144 (1.143, 1.146)^*^	1.445 (1.443, 1.447)^*^	< 0.001
Cardiovascular mortality				
Female	1.00	3.906 (3.885, 3.926)^*^	3.575 (3.556, 3.595)^*^	< 0.001
Male	1.00	0.688 (0.686, 0.691)^*^	1.243 (1.239, 1.247)^*^	< 0.001
Cardio-cerebrovascular mortality				
Female	1.00	3.169 (3.153, 3.184)^*^	2.990 (2.975, 3.006)^*^	< 0.001
Male	1.00	0.723 (0.720, 0.725)^*^	1.084 (1.081, 1.088)^*^	< 0.001

CI confidence interval, HR hazard ratio, SII (Platelet count × Neutrophils)/lymphocytes

Analysis was adjusted for age, gender, ethnicity, education, marital status, smoker, drinker, DM, hypertension, HF, CHD, stroke, cancer, BMI, hemoglobin, glycohemoglobin, platelets, blood urea nitrogen,urea acid, creatinine, cholesterol, glucose, LDL-C, HDL-C, triglyceride, albumin

^*^P < 0.001

**Fig. 4:**
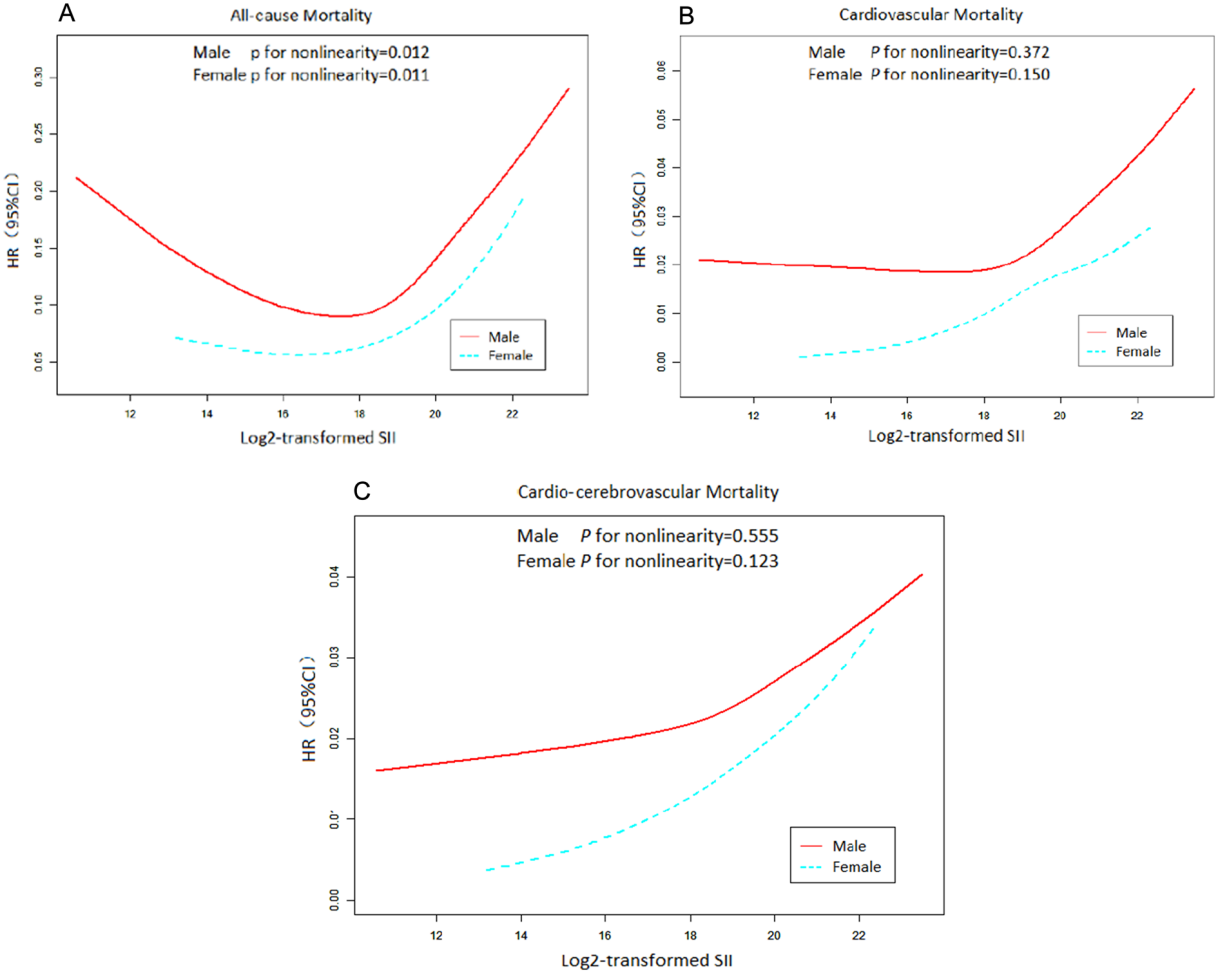
Restricted cubic spline curves of relations between SII and mortality in different sex groups. **A** all-cause mortality; **B** cardiovascular mortality; **C** cardio-cerebrovascular mortality. Analysis was adjusted for age, gender, ethnicity, education, marital status, smoker, drinker, DM, hypertension, HF, CHD, stroke, cancer, BMI, hemoglobin, glycohemoglobin, platelets, blood urea nitrogen, creatinine,uric acid, cholesterol glucose, LDL-C, HDL-C, triglyceride, albumin

Additionally, Fig. 4A shows a nonlinear relationship between the SII and all-cause mortality in women (*P* for nonlinearity = 0.011) and men (*P* for nonlinearity = 0.012). Figure 4B shows a nonlinear relationship between the SII and cardiovascular mortality for women (*P* for nonlinearity = 0.150) and men (*P* for nonlinearity = 0.372). Finally, Fig. 4C indicates that a higher SII was nonlinearly associated with an increased risk of cardio-cerebrovascular mortality in both women (*P* for nonlinearity = 0.123) and men (*P* = 0.555).

## Discussion

This study examined whether the SII could predict long-term outcomes in the general population. Increase in the SII appeared to be significantly related to cardiovascular and cardiocerebrovascular mortality, while it was nonlinearly related to all-cause mortality. The SII was associated with the lowest risk of death at a threshold of 18.28. Similar association patterns were observed in women and men. Although men and women showed comparable associations with all-cause mortality, women exhibited a stronger association with cardiovascular and cardiocerebrovascular mortality. Previous studies have confirmed that sex differences in CVD are attributable to the comprehensive expression of genetic and hormonal differences between men and women [28].

Recently, several studies have reported that the SII can predict CVD's severity, complications, and mortality [29]. Specifically, researchers found that the SII level was an independent prognostic indicator of poor prognosis at 3 months, which was related to the severity of stroke [30]. Furthermore, SII was significantly correlated with cerebral venous thrombosis. Xu et al. [31] reported that SII increased the total stroke risk, while Zhang et al. [32] found a significant correlation between SII and cerebral venous thrombosis. In multiple regression analysis, the degree of SII was a strong independent predictor.

Compared to other biological indicators such as PLR, NLR, or C-reactive protein, SII possesses unique advantages, making it a better predictor of coronary heart disease [33]. The SII is more stable than the blood cell count alone, which can be influenced by dehydration and fluid overload [33, 34]. This study’s results are consistent with previous research demonstrating a positive relationship between SII and cardiovascular mortality. Each twofold increase in the SII resulted in 20.4%, 21.8%, and 16.3% increases in all-cause, cardiovascular, and cardiocerebrovascular mortality, respectively.

The SII was also found to be significantly associated with all-cause mortality in several specific diseases, such as pancreatic [35], oral cavity squamous cell carcinoma [36], lung cancer [37], gastrointestinal cancer [38], urinary system cancer [39]. This study found a significant association between a higher SII and an increased risk of all-cause mortality over an average follow-up period of 88 months.

In part of the curve (SII > 18.28), all-cause mortality increased with increasing SII, and there was a positive correlation between the SII and cardiovascular mortality. This may be because of the significant association between inflammatory and immune status, as reflected by SII and CVD risk. Endothelial activation and platelet coordination are closely related, and recent research has shown a close relationship between cardiovascular mortality, platelet number, and platelet aggregation ability. Combining platelets with fibrin gives rise to a coronary thrombus, which is a crucial player in ACS pathophysiology [40]. In addition to causing blood clots, platelets also deliver mediators contributing to local inflammation [41]. Similarly, neutrophils secrete inflammatory mediators that can cause vessel wall degradation and endothelial dysfunction [42].

In addition, neutrophils interact with platelets, proteolyze coagulation factors, and release prothrombin molecules, activating the inflammatory response [43]. In addition to regulating inflammation, lymphocytes have anti-atherothrombotic properties [44].

Our study provides novel insights by demonstrating a nonlinear association between the SII and all-cause mortality, although the mechanism underlying this association is unclear. A reasonable explanation could be that participants with a lower SII had lower platelet counts. Suppose the platelet count decreases to a certain extent, bleeding symptoms may occur, which could be mild, such as in the skin and mucous membrane areas, or more serious and life-threatening, such as bleeding in the gastrointestinal or intracranial cavities [45, 46]. This may be one of the reasons for the U-shaped relationship between all-cause mortality and the SII.

Furthermore, we observed that the percentages of women and cancer patients in the group with a higher SII were higher, indicating that sex and cancer may explain the increased mortality risk associated with an increase in SII. Further research is needed to confirm the relationship between the SII increase and the risk of all-cause, cardiovascular, and cardio-cerebrovascular mortality.

## Strengths and limitations of the study

Our study, which had a large research population and a long follow-up period, allowed us to better understand the relationship between the SII and all-cause mortality rate of the general population and cardiovascular and cerebrovascular deaths. Furthermore, we used the RCS model to analyze the nonlinear relationship between the SII and all-cause mortality. However, the study has some limitations, such as using self-reported data for complications and living habits, which may have memory bias. Additionally, we only collected the baseline value of the SII, which may not reflect changes in the SII during follow-up. Although we adjusted for many confounding factors, other variables may have influenced the results.

## Conclusions

In summary, this study showed that the SII was independently related to all-cause, cardiovascular, and cardio-cerebrovascular mortality in the general population. These findings suggest that the SII could be useful for identifying individuals at risk of poor clinical outcomes. Considering that the SII is easy and inexpensive to acquire, it could be a convenient tool for clinicians to stratify patient risks and plan preventive and treatment strategies.

### Supplementary Information

**Additional file 1: Table S1.** Baseline characteristics of the included population and the follow-up missing population.

## Data Availability

The data of this study are publicly available on the NHANES website.

## Abbreviations

SII: Systemic immune-inflammation index
NLR: Neutrophil-lymphocyte ratios
PLR: Platelet-lymphocyte ratios
LPM: Laboratory/Medical Technologists Procedures Manual
LDL-C: Low-density lipoprotein cholesterol
HDL-C: High-density lipoprotein cholesterol
ACS: Acute coronary syndrome
NHANES: National Health and Nutrition Examination Survey
NCHS: National Center for Health Statistics
ICD: International Classification of Diseases
DM: Diabetes mellitus
HF: Heart failure
CHD: Coronary heart disease
BMI: Body mass index
HR: Hazard ratio
CI: Confidence interval
RCS: Restricted cubic spline
CAD: Coronary artery disease
NIHSS: National Institutes of Health Stroke Score
