# Supplementary material for: Systemic immune-inflammation index (SII) and the risk of all-cause, cardiovascular, and cardio-cerebrovascular mortality in the general population
Source: Eur J Med Res. 2023 Dec 9;28:575. doi: 10.1186/s40001-023-01529-1 (PMC10709886; doi:10.1186/s40001-023-01529-1)
Supplement: Supplementary file 1 — Additional file 1: Table S1. Baseline characteristics of the included population and the follow-up missing population. [file 40001_2023_1529_MOESM1_ESM.docx]

| Variable  Table S1. Baseline characteristics of the included population and the follow-up missing population | Included population  (n=26,855) | Follow-up missing population  (n=40,246) | *P* value |
| --- | --- | --- | --- |
| SII | 571,361.23 ± 348,108.33 | 524,205.815 ± 350,972.847 | <0.00001 |
| Age，years | 45.674 ± 17.238 | 30.085 ± 22.146 | <0.00001 |
| Male，(%) | 48.268 | 49.650 | 0.00034 |
| Ethnicity, (%)  Non-Hispanic White  Non-Hispanic Black  Mexican American  Other race | 69.179  10.879  8.246  11.697 | 64.220  12.627  10.823  12.330 | <0.00001 |
| Education,(%)  ＜High school  High school  ＞High school  Other | 16.964  22.828  56.628  3.580 | 10.920  13.555  30.800  44.725 | <0.00001 |
| Body mass index, kg/m^2^ | 28.471 ± 6.599 | 24.883 ± 7.252 | <0.00001 |
| Marital status,(%)  Never married  Married  Widowed  other | 20.712  61.746  15.329  2.212 | 17.242  34.671  8.389  39.697 | <0.00001 |
| Drinker, (%) | 11.339 | 7.264 | <0.00001 |
| Smoker, (%) | 45.260 | 26.264 | <0.00001 |
| Diabetes mellitus,(%) | 8.190 | 4.264 | <0.00001 |
| Hypertension, (%) | 29.719 | 15.834 | <0.00001 |
| Heart failure, (%) | 2.323 | 1.181 | <0.00001 |
| Coronary heart disease,(%) | 3.255 | 1.753 | <0.00001 |
| Stroke,(%) | 2.637 | 1.410 | <0.00001 |
| Cancer,(%) | 8.905 | 4.505 | <0.00001 |
| Hemoglobin,g/dL | 14.320 ± 1.500 | 13.975 ± 1.429 | <0.00001 |
| Platelets,10^3^/μL | 254.704 ± 66.015 | 278.239 ± 73.747 | <0.00001 |
| Lymphocyte,10^3^/μL | 2.118 ± 1.018 | 2.403 ± 1.074 | <0.00001 |
| Neutrophil,10^3^/μL | 4.338 ± 1.719 | 4.050 ± 1.721 | <0.00001 |
| Triglyceride, mmol/L | 1.679 ± 1.397 | 1.487 ± 1.032 | <0.00001 |
| Blood urea nitrogen, mmol/L | 4.641 ± 1.901 | 4.446 ± 1.539 | <0.00001 |
| Cholesterol, mmol/L | 5.063 ± 1.063 | 4.800 ± 0.927 | <0.00001 |
| Uric acid, μmol/L | 321.411 ± 83.162 | 313.601 ± 71.280 | <0.00001 |
| Creatinine, μmol/L | 78.541 ± 31.553 | 72.056 ± 27.416 | <0.00001 |
| LDL cholesterol, mmol/L | 2.972 ± 0.905 | 2.740 ± 0.561 | <0.00001 |
| HDL cholesterol, mmol/L | 1.369 ± 0.407 | 1.382 ± 0.311 | 0.00001 |
| Albumin, g/L | 42.789 ± 3.354 | 43.432 ± 3.006 | <0.00001 |
| Glycohemoglobin | 5.554 ± 0.891 | 5.409 ± 0.707 | <0.00001 |

Mean +/- SD for: age, body mass index,hemoglobin,platelet count,albumin,blood urea nitrogen,cholesterol,triglycerides,uric acid,creatine,glycohemoglobin, HDL cholesterol, LDL cholesterol;SII, Lymphocyte; Neutrophil.

P value was calculated by weighted linear regression model.

% for: gender,Ethnicity,education, Marital status, Heart failure, Coronary heart disease, Stroke, Hypertension, cancer,Diabetes mellitus, Drinker, Smoker.

P value was calculated by weighted chi-square test.
